# Comparison of the efficacy of neuronavigation-assisted intracerebral hematoma puncture and drainage with neuroendoscopic hematoma removal in treatment of hypertensive cerebral hemorrhage

**DOI:** 10.1186/s12893-024-02378-3

**Published:** 2024-03-12

**Authors:** Lei Jiang, Jinjie Tian, Chao Guo, Yi Zhang, Ming Qian, Xuejian Wang, Zhifeng Wang, Yang Chen

**Affiliations:** Department of Neurosurgery, Second Affiliated Hospital of Nantong University, Nantong, Jiangsu Province China

**Keywords:** Neuronavigation, Neuroendoscopy, Intracerebral hematoma puncture, Hypertensive cerebral hemorrhage, Hematoma evacuation

## Abstract

**Objective:**

To compare neuronavigation-assisted intracerebral hematoma puncture and drainage with neuroendoscopic hematoma removal for treatment of hypertensive cerebral hemorrhage.

**Method:**

Ninety-one patients with hypertensive cerebral hemorrhage admitted to our neurosurgery department from June 2022 to May 2023 were selected: 47 patients who underwent endoscopic hematoma removal with the aid of neuronavigation in observation Group A and 44 who underwent intracerebral hematoma puncture and drainage in control Group B. The duration of surgery, intraoperative bleeding, hematoma clearance rate, pre- and postoperative GCS score, National Institutes of Health Stroke Scale (NIHSS) score, mRS score and postoperative complications were compared between the two groups.

**Results:**

The duration of surgery, intraoperative bleeding and hematoma clearance were significantly lower in Group B than in Group A (*p* < 0.05). Conversely, no significant differences in the preoperative, 7-day postoperative, 14-day postoperative or 1-month postoperative GCS or NIHSS scores or the posthealing mRS score were observed between Groups A and B. However, the incidence of postoperative complications was significantly greater in Group B than in Group A (*p* < 0.05), with the most significant difference in incidence of intracranial infection (*p* < 0.05).

**Conclusion:**

Both neuronavigation-assisted intracerebral hematoma puncture and drainage and neuroendoscopic hematoma removal are effective at improving the outcome of patients with hypertensive cerebral hemorrhage. The disadvantage of neuronavigation is that the incidence of complications is significantly greater than that of other methods; postoperative care and prevention of complications should be strengthened in clinical practice.

**Supplementary Information:**

The online version contains supplementary material available at 10.1186/s12893-024-02378-3.

## Introduction

Hypertensive cerebral hemorrhage is characterized by high morbidity, high mortality and poor prognosis. Most of these patients experience paralysis, aphasia or other severe disabilities [1]. Its incidence in China is expected to continue to increase over the next decade due to the increasing aging of the population. Early and effective surgical treatment of hypertensive intracerebral hemorrhage (HICH) by removing the intracerebral hematoma can significantly promote functional recovery and reduce the occurrence of complications [2, 3]. With the development of modern imaging technology, neuronavigation technology has been commonly used in surgery [4–7]. Neuroendoscopic intracerebral hematoma removal versus hematoma cavity puncture and drainage is a minimally invasive surgical option for HICH [8, 9]. Herein we discuss the respective advantages of these two techniques with the aid of neuronavigation.

## Materials and methods

### General information

The study was approved by the Ethics Committee of the Second Affiliated Hospital of Nantong University, and all patients provided signed informed consent. Ninety-one patients with HICH admitted to our hospital between June 2022 and May 2023 were selected for the study; all had bleeding in the basal ganglia area. We randomly assigned patients using the following method: an initial sample size of 100 was set, with numbers 1 to 100 representing the admission order of patients who met the inclusion criteria. Randomly sort numbers from 1 to 100 using software. The top 50 will be assigned to Group A (neuroendoscopic group), and the bottom 50 will be assigned to Group B (haematoma puncture group). Nine patients were excluded due to other factors. Group A included 33 males and 14 females, with a mean age of 58.13 ± 12.65 years. Group B included 28 males and 16 females, with a mean age of 62.80 ± 10.76 years. When comparing general information between the two groups of patients, the differences were not statistically significant (*p* > 0.05) and were comparable (Table 1). The inclusion criteria were as follows: (1) meeting clinical HICH diagnostic criteria; (2) hemorrhage located in the basal ganglia and may be associated with ventricular hemorrhage; (3) no brain herniation prior to treatment, with symptoms such as hemiparesis or aphasia; (4) occupying effect hematoma; (5) clear indications for surgery, no obvious contraindications to surgery, signed informed consent for surgery, neuronavigation-assisted hematoma puncture and drainage or neuronavigation-endoscopic hematoma removal surgical treatment. The exclusion criteria were as follows: (1) unstable vital signs; (2) serious medical conditions (cardiac, hepatic or renal dysfunction) and not able to tolerate surgery; (3) cerebrovascular malformations or cranial aneurysms detected by cerebrovascular examination; and (4) significant abnormalities in coagulation function.

**Table 1:** Comparison of general information

Characteristic	Group A(*n* = 47)	Group B(*n* = 44)	x^2^/t	*p*
Sex (n, %)			0.792	0.252
Male	33(70.21)	28(63.64)		
Female	14(29.79)	16(36.36)		
Age (x̅±s, years)	58.13 ± 12.65	62.80 ± 10.76	1.89	0.42
Hypertension class			2.309	0.315
Level 1	15(31.91)	11(25)		
Level 2	25(53.19)	24(54.54)		
Level 3	7(14.90)	9(20.46)		
Onset time (h)	6.28 ± 3.977	5.77 ± 3.124	0.775	0.941
Complications (n, %)			1.272	0.185
Diabetes	10(21.28)	13(29.55)		
Coronary heart disease	1(2.13)	1(2.27)		
Cerebral infarction		1(2.27)		
Amount of cerebral hemorrhage	40.279 ± 8.401	36.345 ± 5.463	2.627	0.059
Entry into the brain chamber (n, %)	21(44.68)	13(29.55)	1.664	0.142
Length of stay in hospital	18.85 ± 9.072	22.36 ± 6.269	2.135	0.307

### Treatment methods

For endoscopic intracerebral hematoma removal with the aid of neuronavigation in Group A patients, the patient’s head was fixed in the cephalic position using a head frame following administration of general anesthesia. The patient’s preoperative CT and MR imaging data were fused with a neuronavigation system to avoid important functional areas and select the closest point of the hematoma to the cortex as the location point. Routine craniotomy was performed with a 2*3 cm bone window, the puncture direction was repositioned by neuronavigation, the sheath was placed at the center of the hematoma, the core was removed, the endoscope was gradually aspirated, and the bleeding was stopped with electrocoagulation if there was considerable active bleeding. A drainage tube was placed, the bone flap was reset after surgery, and the scalp was sutured.

Group B patients were treated with neuronavigation-assisted intracerebral hematoma puncture and drainage. After general anesthesia, the patient’s head was fixed in the head position using a head frame. The patient’s preoperative CT and MR imaging data were fused through the neuronavigation system to avoid important functional areas, and the puncture path was designed according to the location and extent of the hematoma. After the puncture site was selected, the skull was routinely opened, a 1 cm bone hole was drilled, trajectory navigation was used, a drainage tube was placed under the guidance of a navigation stick, the lateral hole of the drainage tube was completely located in the hematoma, the appropriate amount of hematoma was aspirated, an external drainage device was attached, and the scalp was sutured. Postoperative dynamic review of the cranial CT was performed, and in conjunction with the patient’s drainage, urokinase irrigation was used to facilitate liquefaction and drainage of the hematoma if necessary.

### Assessment indicators

The general data collected at admission included age, sex, cerebral hemorrhage volume, whether the hemorrhage entered the ventricles, time of onset, hypertension grade, comorbidities (diabetes, hyperlipidemia), and length of stay. The patient’s clinical parameters were assessed, as follows: operation time, intraoperative bleeding, hematoma clearance rate (preoperative hematoma volume - postoperative hematoma volume)/preoperative hematoma volume*100%, postoperative hematoma volume calculated by the first postoperative CT review in Group A, and postoperative hematoma volume calculated by the first postoperative CT review after drainage tube removal in Group B.

Patient outcomes and healing from surgery were assessed according to the following criteria: NIHSS and GCS scores preoperatively and at 7 days postoperatively, 14 days postoperatively and 1 month postoperatively; and mRS scores postoperatively. The patients were assessed for the following postoperative complications: pulmonary infection, intracranial infection, rebleeding, epilepsy, and lower limb venous thrombosis.

### Statistical analysis

SPSS 23.0 statistical software was used to analyze the data. The data are expressed as x̅±s, and a t test was used for comparisons between groups. The statistical data are expressed as rates or composition ratios, and the χ2 test was used for comparisons between groups. *p* < 0.05 indicated a statistically significant difference.

## Results

### Comparison of patients’ clinical procedures

Compared to Group A, Group B had a shorter mean operative time and less intraoperative bleeding (*p* < 0.001), but a significantly lower hematoma clearance rate (*p* < 0.05) (Table 2).

**Table 2:** Comparison of surgical conditions

Characteristic	Group A(*n* = 47)	Group B(*n* = 44)	t	*P*
Duration of surgery	117.98 ± 33.308	39.66 ± 13.136	14.569	< 0.001
Intraoperative bleeding	288.94 ± 146.528	49.55 ± 21.015	10.731	< 0.001
Hematoma clearance rate	85.803 ± 7.872	72.404 ± 10.771	6.806	0.031

### Comparison of surgical outcomes and healing

After neuroguide-assisted endoscopic hematoma removal in Group A versus hematoma cavity puncture and drainage in Group B, the NIHSS score and GCS score were significantly better postoperatively, with no significant differences in NIHSS score, GCS score or mRS score detected between the groups (Table 3).

**Table 3:** Comparison of surgical outcomes and healing

Characteristic		Group A(*n* = 47)	Group B(*n* = 44)	t	*p*
NIHSS score	Preoperative	20.7 ± 7.407	19.93 ± 5.671	0.554	0.176
	7 days after surgery	17.30 ± 7.031	16.57 ± 6.185	0.390	0.524
	14 days after surgery	13.74 ± 7.094	13.20 ± 5.956	0.392	0.194
	1 month after surgery	11.38 ± 7.067	10.52 ± 6.189	0.439	0.616
GCS score	Preoperative	8.55 ± 2.669	8.61 ± 2.115	0.119	0.058
	7 days after surgery	9.72 ± 2.534	9.70 ± 2.119	0.038	0.309
	14 days after surgery	10.81 ± 2.667	10.70 ± 2.064	0.207	0.074
	1 month after surgery	11.45 ± 2.611	11.59 ± 2.061	0.291	0.123
mRS score				6.199	0.287
	0		4(9.09)		
	1	9(19.15)	13(29.55)		
	2	14(29.79)	10(22.73)		
	3	10(21.28)	9(20.45)		
	4	10(21.28)	5(11.36)		
	5	4(8.51)	3(6.82)		

### Postoperative complications

The incidence of postoperative complications was significantly greater in Group B (*p* < 0.05) than in Group A (*p* < 0.05), with intracranial infection having the most significant incidence (*p* < 0.05), in Group A versus Group B (*p* < 0.05) (Table 4).

**Table 4:** Comparison of postoperative complications

Characteristic	Group A(*n* = 47)	Group B(*n* = 44)	x^2^	*p*
Complications	15(31.91)	27(58.70)	7.964	0.047
Lung infection	8(17.02)	10(22.73)	0.466	0.674
Intracranial infection	3(6.38)	11(25)	6.051	0.014
Secondary bleeding	2(4.26)	5(11.36)	1.617	0.203
Seizure	1(2.13)	1(2.27)	0.02	0.962
Lower limb venous thrombosis	1(2.13)	0	0.947	0.331

## Discussion

Current treatments for HICH include conservative medication and surgery. However, surgical management of HICH is controversial, and there is no uniform standard for indications, timing or choice of approach. Nevertheless, for supratentorial hematoma > 30 mL with significant peri-hematoma edema and midline shift and for subratentorial hematoma > 10 mL with significant intracranial hypertension and cerebellar symptoms, impaired consciousness and progressive worsening of the condition may occur. Brain herniation after a brain hemorrhage with dilated pupils on one side and loss of response to light, intracerebroventricular hematoma or presence of obstructive hydrocephalus should be treated with aggressive surgical procedures [10–12]. The main objective of surgery is to relieve the pressure of an intracerebral hematoma on surrounding brain tissue, relieve intracranial hypertension, minimize secondary brain damage caused by hematoma compression, save patients’ lives and improve patients’ healing and quality of life. Conventional surgical methods include neuroendoscopic intracerebral hematoma removal, intracerebral hematoma puncture and drainage, and microscopic craniotomy hematoma removal, all of which have advantages and disadvantages.

**Fig. 1:**
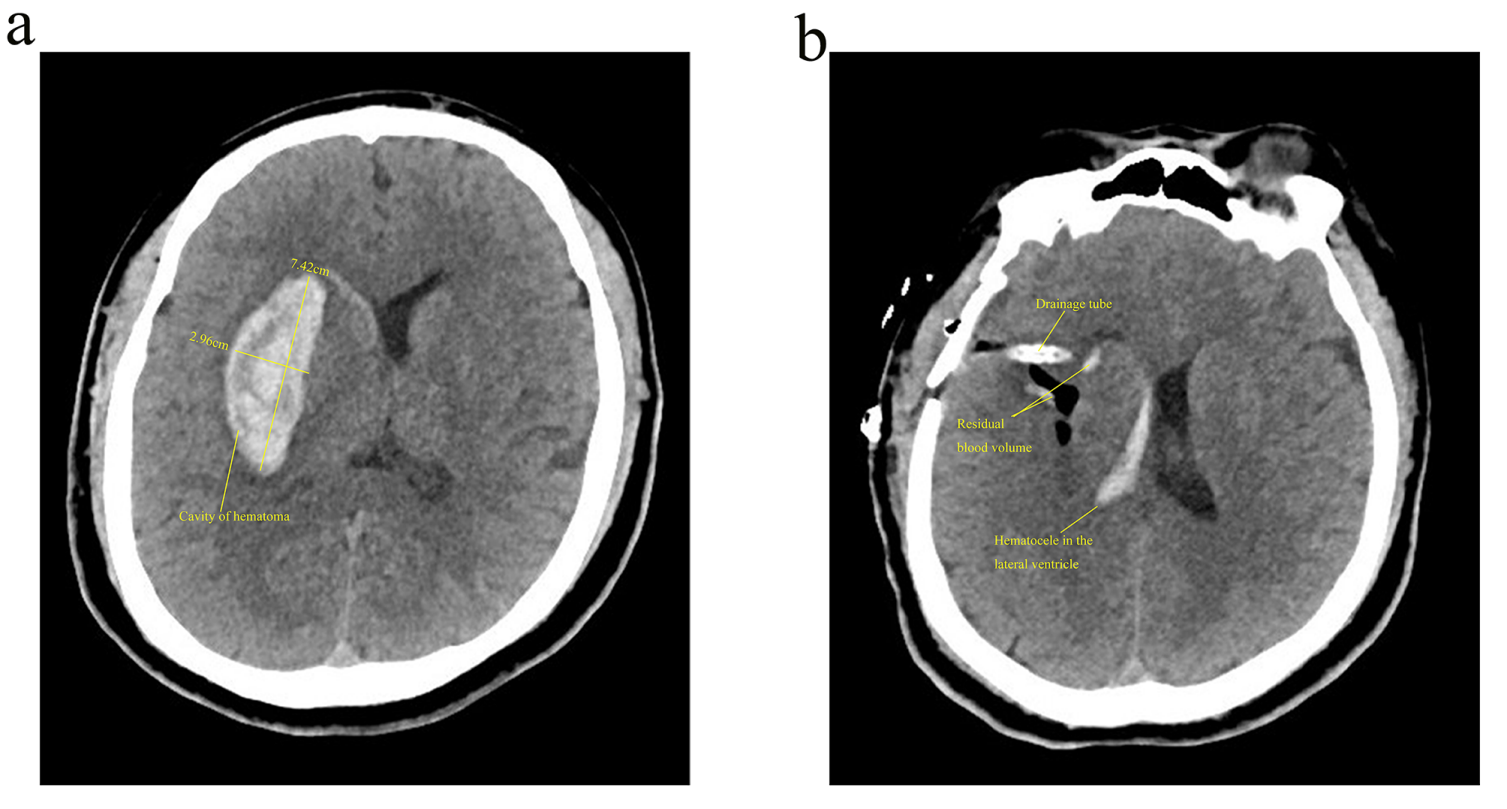
A 56-year-old woman experienced right hemiplegia for 3.5 h and underwent emergency neuroendoscopic removal of cerebral hematoma. (**a**) Preoperative CT, hematoma volume 36.724 ml; (**b**) Postoperative CT, remaining hematoma volume 2.031 ml

**Fig. 2:**
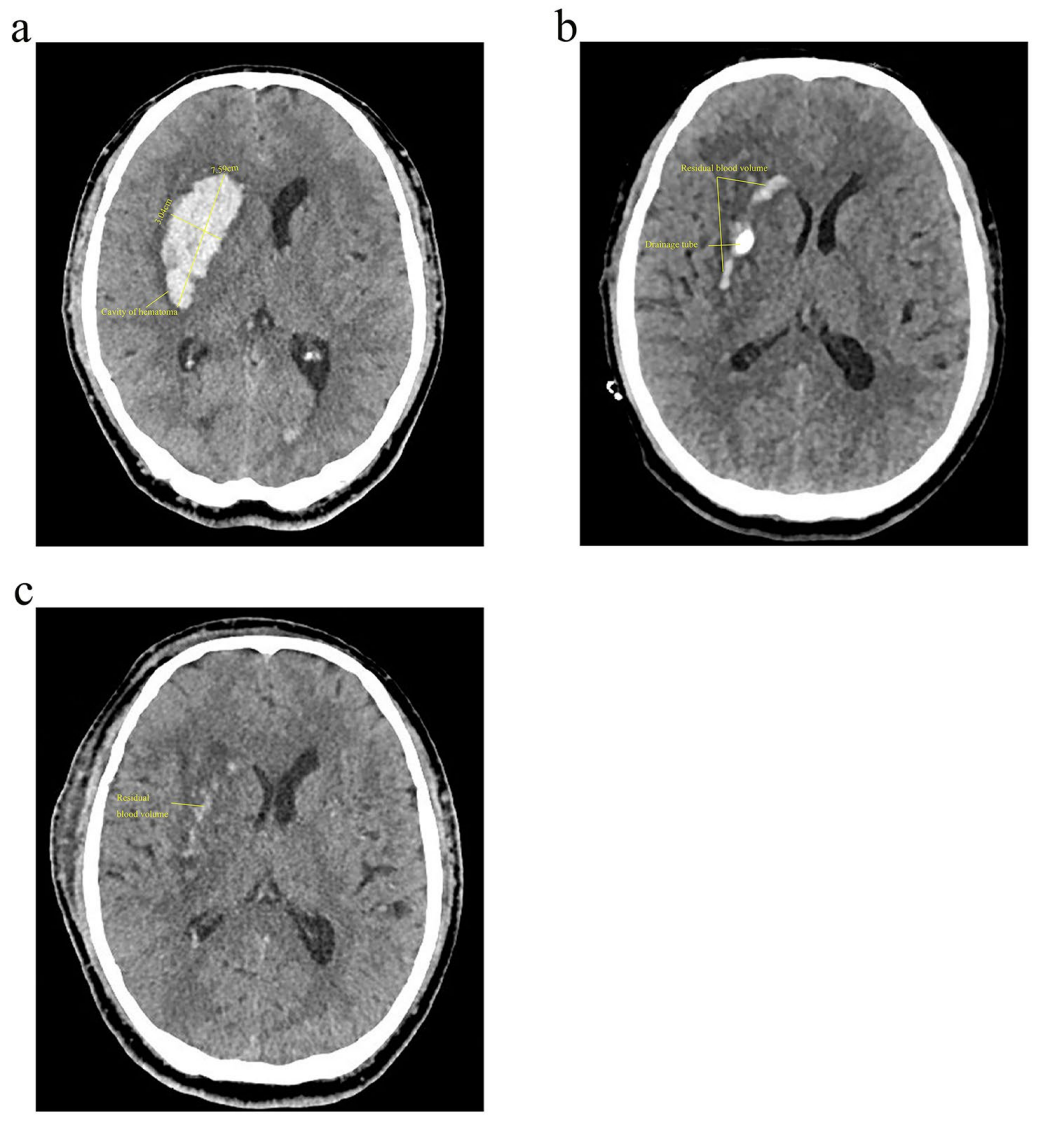
A 64-year-old man experienced headache for 3 h and underwent neuronavigation-assisted intracerebral hematoma puncture and drainage of cerebral hematoma. (**a**) Preoperative CT, hematoma volume 33.701 ml; (**b**) CT before extubation, remaining hematoma volume 5.762 ml; (**c**) CT after extubation, remaining hematoma volume 1.935 ml

In recent years, neuronavigation has reduced the incidence of surgical complications by integrating computer technology and neuroimaging technology to design an optimal surgical approach, accurately locating bleeding foci and reducing brain tissue damage to important structures. As a result, this technique has been widely used in clinical management of HICH in recent years and has greatly improved patient prognosis [13, 14]. Neuroguided endoscopic hematoma removal is also becoming more accepted by neurosurgeons as a mainstream procedure. It is less invasive and allows for visualization of the hematoma and hemostasis and complete removal of the hematoma but requires a certain level of operator skill [15–18] (Fig. 1a and b). Moreover, intracerebral hematoma puncture and drainage through use of neuronavigation equipment, application of local thrombolytic agents after establishment of surgical access, local monitoring and other means have been developed in the study of minimally invasive diagnosis and treatment of cerebral hemorrhage [19, 20] (Fig. 2a, b and c).

To provide neurosurgeons with a reference for indications for surgery, surgical approach, postoperative management, and prevention and treatment of complications in patients with cerebral hemorrhage, this study involved an analysis of patients with hypertensive cerebral hemorrhage admitted to our hospital from June 2022 to May 2023. The results showed no significant difference between neuronavigation-assisted hematoma puncture and drainage or endoscopic hematoma removal in terms of patient outcome. However, the duration of the procedure and intraoperative bleeding in the hematoma puncture group were significantly less than those in the endoscopic group, whereas the rate of hematoma removal was reduced compared to the endoscopic group. The rate of postoperative complications, especially intracranial infections, was significantly greater in the hematoma puncture group than in the endoscopic group. In response to these results, we provide the following discussion. Firstly, neuronavigation offers greater convenience than endoscopic intracerebral hematoma removal for hematoma puncture and drainage. Hematoma puncture itself is easy to perform, as the preoperative 3D display of an intracranial hematoma allows for the design of the puncture route, avoiding blind puncture damage while better ensuring placement of the drainage tube in the hematoma [21–24]; this reduces the length of the operation even more and reduces intraoperative bleeding. The hematoma clearance rate in the endoscopic group is determined by comparing the preoperative CT hematoma volume with the remaining hematoma volume on the first postoperative CT review, and the hematoma clearance rate in the puncture group is determined by comparing the preoperative CT hematoma volume with the remaining hematoma volume on the first postoperative CT review after the drainage tube is removed. Although drainage tube removal is influenced by the duration of drainage tube retention, invasive tube infection, and the amount of hematoma remaining on postoperative review CT, absorption of hematoma remaining after drainage tube removal in the puncture group and in the endoscopic group after surgery was independent of operative interference, and the group comparison of the hematoma clearance rate on the effect of healing was meaningful. As we know, most patients with cerebral hemorrhage in the basal ganglia have hemiplegia, long-term bed rest is required after surgery. And the average length of stay was significantly greater in the puncture group (22.36 ± 6.269 days) than in the endoscopic group (18.85 ± 9.072 days), with a significantly greater rate of Lung infection in those who were bedridden for longer periods after the procedure. We consider that this issue is not only related to long-term bed rest and postoperative care for patients but also to the use of artificial airways after surgery. We will further focus on this issue in the future. The longer duration of intracranial drainage in the puncture group and the need for urokinase flushing in most patients increases the risk of intracranial infection compared to the endoscopic group [25].

In conclusion, both neuronavigation-assisted intracerebral hematoma puncture and drainage and neuroendoscopic hematoma removal are effective at removing intracerebral hematomas and improving the outcome of patients with hypertensive cerebral hemorrhage. Although neuronavigation has made intracerebral hematoma puncture and drainage more convenient during surgery, the disadvantage is that the incidence of complications is significantly greater than that of other methods, and postoperative care and prevention of complications should be strengthened in clinical practice.

We compared our research with existing literature. Masahito [26] compared endoscopic surgery with craniotomy and concluded that while endoscopic surgery shortened the surgical time, it did not have a significant impact on the postoperative improvement of patients. Wu [27] believes that the combination of endoscopic surgery and ICP monitoring may improve the clinical efficacy and treatment outcomes of HICH patients. It is difficult to directly compare our results with the studies of Scaggiante and Wu. However, we can still observe that while the choice of surgical methods may not have a significant impact on the improvement of patient prognosis, there are indeed some patients with cerebral hemorrhage who ultimately benefit from surgery.

This was a randomized controlled clinical study with a small number of patients and a short follow-up observation period during the ambulatory period, and multicenter studies are needed for comparative validation. The impact of preoperative hemostasis, prevention of vascular spasm, and other medications on surgical outcomes was not included in the evaluation. Even if the surgical physicians remain the same, various anesthesia teams and postoperative care teams can be potential confounding factors that may impact the surgery and prognosis.

### Electronic supplementary material

Below is the link to the electronic supplementary material.

Supplementary Material 1

## Data Availability

The datasets created and/or analyzed during the current study are available from the corresponding author upon reasonable request.
