# Supplementary material for: Comparison of the efficacy of neuronavigation-assisted intracerebral hematoma puncture and drainage with neuroendoscopic hematoma removal in treatment of hypertensive cerebral hemorrhage
Source: BMC Surg. 2024 Mar 12;24:86. doi: 10.1186/s12893-024-02378-3 (PMC10935852; doi:10.1186/s12893-024-02378-3)
Supplement: Supplementary file 1 — Supplementary Material 1 [file 12893_2024_2378_MOESM1_ESM.pdf]

STROBE Statement

	Item No	Recommendation
**Title and abstract**	1	(*a*) Comparison of the efficacy of neuronavigation-assisted intracerebral hematoma puncture and drainage with neuroendoscopic hematoma removal in treatment of hypertensive cerebral hemorrhage
		(*b*) A total of 91 patients with hypertensive intracerebral hemorrhage were selected. The operation time, intraoperative blood loss, hematoma clearance rate, preoperative and postoperative GCS score, NIHSS score, mRS Score and postoperative complications were compared between the two groups. We found that both neuronavigation-assisted intracerebral hematoma puncture and drainage and neuroendoscopic hematoma removal are effective in improving the outcome of patients with hypertensive cerebral hemorrhage. The disadvantage of neuronavigation is that the incidence of complications is significantly greater than that of other methods; postoperative care and prevention of complications should be strengthened in clinical practice.
**Introduction**		
Background/rationale	2	Hypertensive intracerebral hemorrhage has the characteristics of high incidence, high mortality and poor prognosis.
Objectives	3	Explore the respective advantages of both techniques with the aid of neural navigation
**Methods**		
Study design	4	Prospective cohort study
Setting	5	Ninety-one patients with HICH admitted to our hospital from June 2022 to May 2023 were selected as the study objects, and the bleeding sites were all located in the basal ganglia region. According to the different clinical treatment methods, they were divided into two groups: A neuroendoscopy group and B hematoma puncture group.
Participants	6	(*a*) Ninety-one patients with HICH admitted to our hospital from June 2022 to May 2023.
		(*b*) The patients were divided into two groups according to their clinical treatment modalities: A, the neuroendoscopic group; and B, the hematoma puncture group. Group A included 33 males and 14 females, with a mean age of 58.13 ± 12.65 years. Group B included 28 males and 16 females, with a mean age of 62.80 ± 10.76 years.
Variables	7	a.Grouped according to the type of surgery.<br>b. Condition of hospitalization.<br>c. Clinical operation.<br>d. Operative effect and recovery.<br>e. Postoperative complications.
Data sources/ measurement	8*	*The duration of surgery, intraoperative bleeding, haematoma clearance rate, pre- and post-operative GCS score, National Institutes of Health Stroke Scale (NIHSS) score, mRS score and post-operative complications were compared between the two groups.*
Bias	9	SPSS 23.0 statistical software was applied to analyse the data, The data are expressed as x±s, and t-test was used for comparison between groups. Statistical data are expressed as rates or composition ratios, and the $\chi 2$ test was used for comparison between groups. $p<0.05$ for statistically significant difference.
Study size	10	Hypertensive cerebral hemorrhage surgery performed by the same group of neurosurgeons within one year.
Quantitative variables	11	None
Statistical methods	12	SPSS 23.0 statistical software was applied to analyse the data, The data are expressed as x±s, and t-test was used for comparison between groups. Statistical data are

		expressed as rates or composition ratios, and the $\chi 2$ test was used for comparison between groups. $p<0.05$ for statistically significant difference.
**Results**		
Participants	13*	A total of 91 people were included in the study, and all patients completed follow-up and analysis.
Descriptive data	14*	All the included researchers were patients with cerebral hemorrhage admitted to our hospital, and the bleeding site was located in the basal ganglia region.
Outcome data	15*	Clinical data of 91patients with hypertensive cerebral haemorrhage admitted to our neurosurgery department from June 2022 to May 2023
Main results	16	(*a*) Endoscopic hematoma removal with the aid of neuronavigation compared to group A, Group B had a shorter mean operative time and less intraoperative bleeding ($p<0.001$), but a significantly lower hematoma clearance rate ($p<0.05$) (Tab 2).
		(*b*) Neuroguide-assisted endoscopic haematoma removal in group A versus hematoma cavity puncture and drainage in group B, NIHSS scores and GCS scores were significantly better in all groups postoperatively, but no significant differences in NIHSS scores, GCS scores and mRS scores were demonstrated between groups (Tab 3).
		(*c*) The incidence of postoperative complications was significantly higher in group B ($p<0.05$) compared to group A ($p<0.05$), with the most significant incidence of intracranial infection ($p<0.05$), when endoscopic hematoma removal was performed with the aid of neuronavigation in group A versus group B ($p<0.05$) (Tab 4).
Other analyses	17	None
**Discussion**		
Key results	18	Neuronavigation has made intracerebral haematoma puncture and drainage more convenient during the operation, while the disadvantage is that the incidence of complications is significantly higher, and postoperative care and prevention of complications should be strengthened in clinical practice.
Limitations	19	This study is a retrospective clinical study with a small number of cases and a short follow-up observation period during the ambulatory period, and a large number of multicentre studies are still needed for comparative validation.
Interpretation	20	Conventional surgical methods include neuroendoscopic intracerebral hematoma removal, intracerebral hematoma puncture and drainage, and microscopic craniotomy hematoma removal, all of which have their own advantages and disadvantages.
Generalisability	21	The purpose of this study is to provide a reference for neurosurgeons regarding surgical indications, selection of surgical methods, postoperative management, and prevention and treatment of complications for patients with cerebral hemorrhage.
**Other information**		
Funding	22	the work was supported by the Science and Technology Support Program of Nantong (JC12022015) and Scientific Research Foundation of Nantong Health Committee (MB2021026) and 'Top Six Types of Talents' Financial Assistance of Jiangsu Province Grant (WSW-199).

*Give information separately for exposed and unexposed groups.
